# Prevalence and Pattern of Internet Addiction Among Adolescents in Ibadan, Nigeria: A Cross-Sectional Study

**DOI:** 10.7759/cureus.22293

**Published:** 2022-02-16

**Authors:** Aanuoluwapo A Afolabi, Olayinka S Ilesanmi, Ayodeji M Adebayo

**Affiliations:** 1 Medicine, University of Ibadan, Ibadan, NGA; 2 Community Medicine/Infectious Disease Epidemiology, University of Ibadan/University College Hospital, Ibadan, NGA; 3 Reproductive and Family Health, University of Ibadan/University College Hospital, Ibadan, NGA

**Keywords:** nigeria, adolescent health, mental health, internet, addiction, adolescent

## Abstract

Background and objective

Despite the potential benefits the Internet offers, it is prone to excessive and uncontrolled use, thus resulting in a condition called Internet addiction (IA). This study aimed to describe the prevalence of Internet addiction (IA) among in-school adolescents in Ibadan, Southwest Nigeria.

Materials and methods

A descriptive cross-sectional study was conducted among 632 adolescents using a two-stage sampling technique. IA was assessed using the 20-question Internet addiction test (IAT). Responses to each question ranged from “0” (i.e., “never”) to “5” (i.e., “always”). Cumulative IAT scores > 50% suggested the presence of IA. Chi-square tests were conducted to determine the association between adolescents’ characteristics and IA. Statistically significant variables were pooled into the binary logistic regression model. P-values < 0.05 were statistically significant.

Results

The mean age of the adolescents was 16.03 ± 1.26 years, and 347 (54.9%) were males. A total of 284 (44.9%) adolescents had IA: 174 (42%) accessed the Internet in both home and school settings (ᵡ^2^ = 4.103, p = 0.043), and 174 (42%) accessed the Internet at home only (ᵡ^2^ = 5.003, p = 0.025). Adolescents who accessed the Internet from both home and school settings had higher odds of developing IA (adjusted odds ratio (AOR) = 1.408, 95% confidence interval (CI) = 0.986-2.012, p = 0.060), as well as those who accessed the Internet from home settings only (AOR = 1.404, 95%CI = 1.010-1.953, p = 0.043). Adolescents who gained four to six hours of Internet connection weekly had two times odds of developing IA (AOR = 1.404, 95%CI = 1.010-1.953, p = 0.043), and those who gained more than six hours of Internet connection had more than three times odds of developing IA (AOR = 3.424, 95%CI = 1.937-6.053, p = 0.043).

Conclusion

To prevent IA, adolescents should develop self-control skills and self-regulation of Internet use. Likewise, Internet access should be restricted from both home and school settings, and adolescents’ Internet access should be monitored and regulated from both home and school settings.

## Introduction

Despite the potential benefits the Internet offers, it is prone to excessive and uncontrolled use, thus resulting in a condition called Internet addiction (IA) [1]. IA disorder is defined as too much computer use that contradicts daily activities in ways that harm daily function [2]. Many pieces of literature have reported a strong association between the onset of IA and the duration spent on the Internet. Nakayama et al. defined IA as weekly Internet use that exceeds 12 hours [3]. A study conducted in Turkey reported that the daily use of the Internet for over two hours is related to IA and mental disorders [4].

IA can occur among all age groups; however, adolescents are particularly vulnerable to IA because of the mental, emotional, and social developments associated with this period [5]. IA has generally been strongly associated with reduced sleeping time, a tendency for postponing sleep, insomnia, excessive tiredness, anxiety, depression, suicide, and attention deficit hyperactivity disorder [6-8]. In lieu of this, the Australian government has developed guidelines for a maximum screen time of two hours daily for schoolchildren and adolescents [9]. Also, the Chinese government has begun to ban the opening of new Internet cafes as a government campaign to clamp down on IA. They have also banned the entry of children below 16 years to Internet cafes to improve students’ concentration on academic work [10].

Many studies have been conducted to identify the home as a risk factor for the development of IA among in-school adolescents [5-8]. However, there is limited literature that assesses Internet exposure in schools as a risk factor for IA among adolescents. In addition, the underlying factors responsible for the outplay of IA among these adolescents are relatively unknown. Neglect of IA would result in an increased vulnerability of adolescents to IA and related disorders, thereby resulting in comorbidities for which more healthcare costs would be incurred on their treatment on the family and nation. A study in this regard would be helpful in planning appropriate interventions among present-day adolescents to ensure that their physical and mental health is not adversely affected. This study, therefore, aimed to describe the pattern (access type, access location, frequency, and motive) of Internet use, as well as the prevalence and factors associated with IA among in-school adolescents in Ibadan, Southwest Nigeria.

## Materials and methods

Study design and area

This was a descriptive cross-sectional study conducted in Ibadan. Ibadan is the capital city of Oyo state and the third-largest city in Nigeria using population projections from the 2006 Nigerian population census [11]. Ibadan is located 7°23′47″N and 3°55′0″E, and it has a total area of 3,080 km^2^ [12]. It is 128 km northeast of Lagos and 345 km southwest of Abuja, the Federal Capital Territory [12]. Internet service providers such as Global Communications Limited and Mobile Telecommunications Network have improved Internet access through the erection of masts in public areas [13].

Study population and sample size

The study was conducted among currently enrolled high school students in selected public secondary schools in Ibadan Southeast Local Government Area (LGA) between April 29 and May 3, 2021, shortly after the suspension of school closure in Nigeria due to the COVID-19 pandemic. Public schools are composed of a homogeneous population of students.

The sample size of in-school adolescents was determined using the sample size formula for cross-sectional studies [14]. A prevalence of 32% [15] was used at a 99% confidence interval (CI) and an allowable error of 4.5%. Thus, the minimum sample size obtained after sample size calculation was 675 in-school adolescents.

Sampling technique and data collection

The selection criterion for the study was the possession of computer laboratories in schools. A two-stage sampling technique was used to enroll participants.

Stage One

From the list of schools obtained from the Zonal Chairman of the Teaching Service Commission, three (one boys-only, one girls-only, and one mixed) schools were selected out of the eight schools with computer laboratories using simple random sampling (balloting method).

Stage Two

Of the 12 arms present in each school, four classes were selected from each through a simple random sampling technique (balloting method).

A semi-structured interviewer-administered questionnaire was used for data collection. The questionnaire was divided into four sections: section A: sociodemographic characteristics, which included age, sex, school, family type, parent’s educational qualification, parent’s age, and parent’s occupation; section B: pattern of Internet use, which included questions on the current ownership of an Internet-enabled device, type of Internet-enabled device(s) owned, the motive for Internet access, the pattern of Internet social network type ever used, site(s) of Internet connection, the pattern of restriction to Internet access, and the duration of Internet connection (during school hours and during weekends); section C: causes, effects, and preventive measures for Internet addiction, in which one structured question was used to elicit information on the causes of IA (including boredom, loneliness, poor physical relationships, and idleness), one structured question was used to elicit information on the effects of IA, and one structured questionnaire was used to elicit information on the preventive measures for IA; and section D: Internet addiction test (IAT), an addiction test comprising 20 questions [16]. The IAT was used to determine the prevalence of IA among adolescents. IAT scores were computed with “0” assigned for “never happened,” “1” for “once weekly,” “2” for “twice weekly,” “3” for “three times weekly,” “4” for “four times weekly,” and “5” for “five times weekly or more.”

Statistical analysis

The collected data were input into the computer daily. Data were analyzed using SPSS version 25.0 (IBM Corporation, Armonk, NY, USA) [17]. Descriptive statistics were summarized using frequency tables, percentages, mean, standard deviation, and charts. The number of hours with daily Internet access and age of onset of Internet use were computed using mean and standard deviation. The average time spent online per day during school hours and after school hours on weekdays and during weekends was computed using frequencies and percentages. The overall average time spent on the Internet (either at home or in school) was categorized into three: “three hours or less,” “four to six hours,” and “more than six hours.”

For the IAT, the cumulative score in the 20 domains yielded a maximum score of 100 points. IAT scores ranging between 20 and 49 points suggested complete control over Internet use. A range of scores between 50 and 70 points was suggestive of occasional IA, while IAT scores greater than 70 points implied significant IA. Occasional and significant IA were categorized together as the presence of IA, while healthy (complete control over) Internet use was categorized as the absence of IA.

Chi-square tests were used to determine the association between sociodemographic characteristics and IA. Variables that were statistically significant at a 5% level of significance in the bivariate analysis were pooled into the binary logistic regression, which was conducted (on variables such as age, school, the current ownership of an Internet-enabled device, Internet connection (at home only, in school only, or both at home and in school settings), self-perception of problematic Internet use, and average time spent on the Internet weekly) to determine the predictors of IA.

Ethics

Ethical approval for this study was obtained from the Oyo State Research Ethics Committee (AD 13/479/4130A). Permission to conduct the study was obtained from the Deputy Administrator of each school. Assent was obtained from adolescents below 18 years, while consent was obtained from those aged 18 years or older. The adolescents were informed of the right to opt-out of the study before the administration of the questionnaire. Participation in this study did not expose adolescents to any known form of harm or injury; rather, the study was helpful in the identification of IA among adolescents as a step toward influencing healthy Internet use habits among them.

## Results

Sociodemographic characteristics

A total of 632 questionnaires were filled out of the 675 questionnaires distributed. Thus, a response rate of 93.6% was obtained.

The mean age of the 632 in-school adolescents was 16.03 ± 1.26 years. Among them, 433 (68.5%) were older than 15 years; 324 (51.3%) were enrolled at a mixed school, and 162 (25.6%) were enrolled in a girls-only school (Table 1).

**Table 1:** Sociodemographic characteristics of in-school adolescents in Ibadan Southeast LGA, 2021

Variables	Frequency	%
Age group (years)		
≤15	199	31.5
>15	433	68.5
Sex		
Male	347	54.9
Female	285	45.1
School		
Boys’ only	146	23.1
Girls’ only	162	25.6
Mixed	324	51.3
Family type		
Nuclear	491	77.7
Extended	98	15.5
No response	43	6.8

Among the adolescents, 216 (41.9%) commenced Internet use at 12 years or below at a mean age of 12.67 ± 2.3 years. Overall, 433 (68.2%) in-school adolescents reported current ownership of Internet-enabled devices. Among them, 374 (86.8%) owned a smartphone, 42 (9.7%) owned a laptop, and 17 (3.9%) owned a tablet.

Pattern of restriction placed on adolescents’ Internet access

Pattern of Restriction of Internet Access From Home and School Settings

In total, 208 (28.2%) adolescents regularly gained Internet access from home settings only, while 414 (71.8%) gained Internet access from both home and school settings. All parents (100%) were aware of their adolescents’ Internet access. However, 370 (58.5%) adolescents had restricted Internet access from home settings only; 257 (69.5%) parents put regular checks on adolescents’ browsing devices, 98 (26.5%) parents frequently reminded adolescents to be careful of some Internet sites, 57 (15.4%) parents strictly monitored adolescent’s Internet use, and 31 (8.4%) parents fixed specific browsing schedule. On the other hand, 75 (11.9%) had restrictions to Internet access from both home and school settings. However, 147 (23.3%) had restrictions to Internet access in school settings only. While connecting to the Internet in the computer laboratory, 72 (49%) teachers regularly checked adolescents’ browsing devices, 51 (34.7%) frequently reminded adolescents to be wary of some Internet sites, 25 (17%) strictly monitored adolescent’s Internet use, and 15 (10.2%) fixed specific browsing schedule.

Motives for Internet access

**Figure 1:**
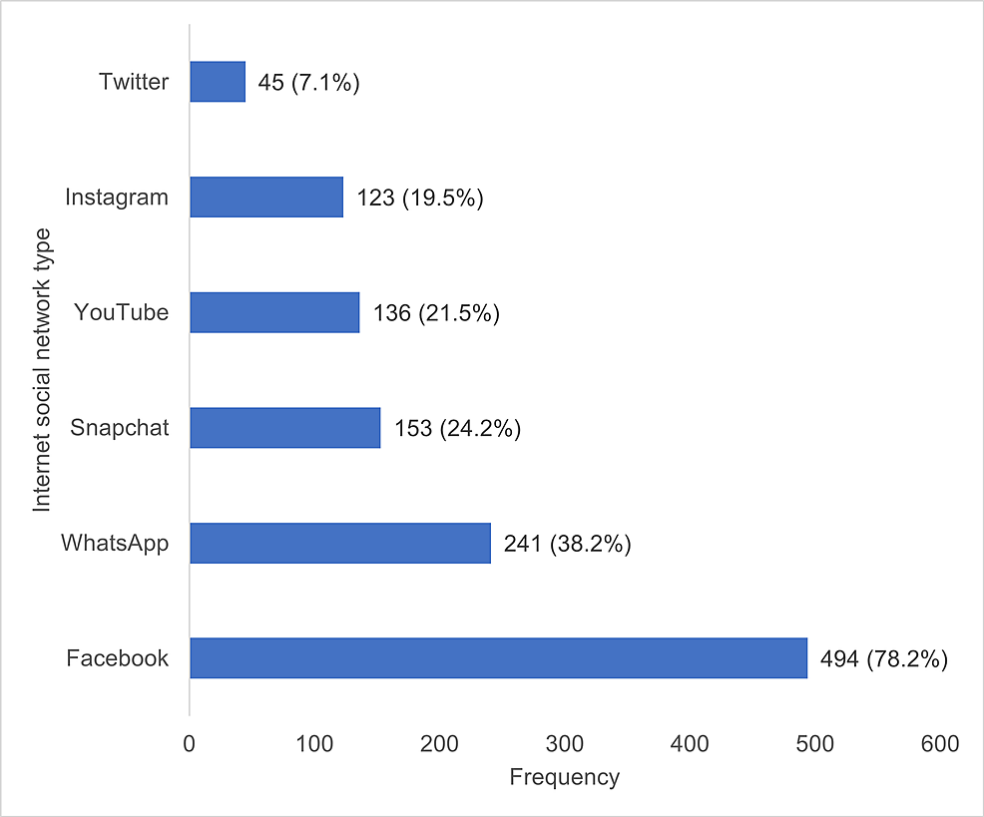
Pattern of use of Internet-enabled devices among in-school adolescents in Ibadan, Nigeria

Regarding the pattern of Internet social network type used by the adolescents, 494 (78.2%) had used Facebook, 241 (38.2%) had accessed WhatsApp, and 153 (24.2%) had made use of Snapchat (Figure 1).

Pattern of Internet access in the past one week among adolescents

Overall, 334 (52.8%) adolescents had accessed the Internet in the past one week in school settings only through the computer laboratory. Among them, 184 (55.1%) had spent one hour or less, and 87 (26%) had spent between two and three hours online. A total of 414 (65.5%) adolescents had accessed the Internet both at home and in school in the past one week. Among them, 223 (53.9%) had spent one hour or less on the Internet at home after school hours (Monday to Friday), and 166 (40.1%) had spent one hour or less on the Internet at home during the weekend (Table 2).

**Table 2:** Frequency of Internet access from school and home settings among in-school adolescents in Ibadan, 2021

Variables	Frequency	%
Accessed the Internet in the school only through the computer laboratory in the past one week		
Yes	334	52.8
No	298	47.2
Average time spent on the Internet in the school in the past one week (Monday to Friday combined) (hours) (n = 334)		
≤1	184	55.1
2–3	87	26
>3	63	18.9
Accessed the Internet at home only in the past one week		
Yes	414	65.5
No	218	34.5
Average time spent on the Internet at home after school hours (Monday to Friday) in the past one week (n = 414)		
≤1	223	53.9
2–3	90	21.7
>3	101	24.4
Average time spent on the Internet at home during the weekend (Saturday and Sunday) in the past one week (hours) (n = 414)		
≤1	166	40.1
2–3	118	28.5
>3	130	31.4
Accessed the Internet in home and school settings		
Yes	414	65.5
No	218	34.5
Overall average time spent on the Internet weekly at home and school (hours) (n = 414)		
≤3	168	40.6
4–6	175	42.3
>6	71	17.1

Effects of and preventive strategies for Internet addiction

Overall, 623 (98.6%) reported that IA could result in procrastination, 622 (98.2%) mentioned that IA could result in anxiety, and 619 (97.9%) stated that IA could result in family disconnectedness. Other effects of IA mentioned included reduced work productivity among 616 (97.5%), depression among 614 (97.2%), loss of sleep among 610 (96.5%), and reduced academic performance among 608 (96.2%).

**Figure 2:**
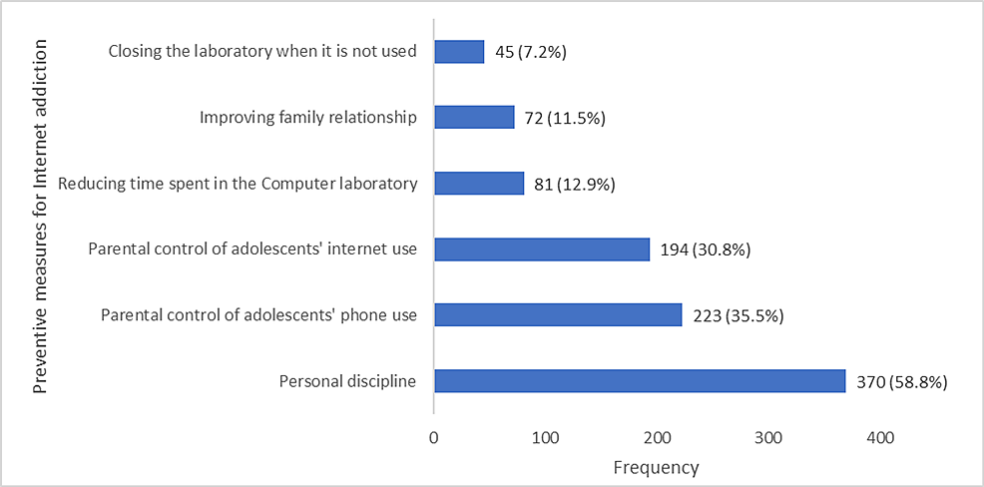
Self-reported strategies for preventing Internet addiction among in-school adolescents in Ibadan, Nigeria

Regarding the preventive measures for IA among the general population of adolescents interviewed, 370 (58.5%) stated personal discipline, 223 (35.3%) mentioned parental control of adolescent’s phone use, and 194 (30.7%) stated parental control of adolescent’s Internet use (Figure 2).

Association between Internet addiction and sociodemographic characteristics of adolescents

IA was present among 78 (39.2%) adolescents aged 15 years or below compared with 206 (47.6%) above 15 years (ᵡ^2^ = 3.869, p = 0.049). Forty-seven (32.2%) in-school adolescents in the boys-only school showed presence of IA compared with 80 (49.4%) in girls-only school and 157 (48.5%) in mixed school (ᵡ^2^ = 12.501, p = 0.002) (Table 3).

**Table 3:** Association between the pattern of Internet access and internet addiction among in-school adolescents in Ibadan Southeast LGA, 2021

Variables	Internet addiction		ᵡ^2^	p-value
	Present, n (%)	Absent, n (%)		
Age of onset of Internet use				
≤12	108 (50)	108 (50)	1.620	0.203
>12	133 (44.3)	167 (55.7)		
Currently own an Internet-enabled device				
Yes	206 (47.8)	225 (52.2)	4.477	0.034
No	78 (38.8)	123 (61.2)		
Accessed the Internet in the school only through the computer laboratory in the past one week				
Yes	178 (53.3)	156 (46.7)	19.992	<0.001
No	106 (35.6)	192 (64.4)		
Average time spent on the Internet in the school in the past one week (Monday to Friday combined) (hours) (n = 334)				
≤1	93 (50.5)	91 (49.5)	4.137	0.042
2–3	45 (51.7)	42 (48.3)		
>3	43 (68.3)	20 (31.7)		
Accessed the Internet at home only in the past one week				
Yes	174 (42)	240 (58)	5.003	0.025
No	112 (51.4)	106 (48.6)		
Average time spent on the Internet at home after school hours (Mondays to Fridays) in the past one week (n = 414)				
≤1	67 (30)	156 (70)	29.500	<0.001
2–3	47 (52.2)	43 (47.8)		
>3	60 (59.4)	41 (40.6)		
Average time spent on the Internet at home during the weekend (Saturday and Sunday) in the past one week (hours) (n = 414)				
≤1	51 (30.7)	115 (69.3)	20.558	<0.001
2–3	49 (61.5)	69 (58.5)		
>3	74 (56.9)	56 (43.1)		
Accessed the Internet in home and school settings				
Yes	174 (42)	240 (58)	4.103	0.043
No	110 (50.5)	108 (49.5)		
Overall average time spent on the Internet weekly at home and school (hours) (n = 414)				
≤3	47 (28)	121 (72)	31.613	<0.001
4–6	80 (45.7)	95 (54.3)		
>6	47 (66.2)	24 (33.8)		

Association between the restrictions implemented by parents/guardians and teachers and adolescents’ Internet addiction

A total of 165 (44.6%) in-school adolescents whose Internet use was restricted at home by their parents had IA compared with 119 (45.4%) whose Internet use was not restricted by parents (ᵡ^2^ = 1.423, p = 0.233). Also, 83 (51.2%) of in-school adolescents who had accessed the Internet from both school and home settings had IA compared with 201 (42.8%) who had not accessed the Internet from both settings (ᵡ^2^ = 3.492, p = 0.062). Further, 130 (62.5%) of those who perceived they had IA manifested IA compared with 154 (36.6%) who perceived otherwise (ᵡ^2^ = 37.770, p ≤ 0.001) (Table 4).

**Table 4:** Association between the restrictions implemented by parents/guardians and teachers and Internet addiction among in-school adolescents in Ibadan Southeast LGA, 2021

Variables	Internet addiction		ᵡ^2^	p-value
	Present, n (%)	Absent, n (%)		
Restriction of Internet access at home only				
Yes	165 (44.6)	205 (55.4)	0.042	0.837
No	119 (45.4)	143 (54.6)		
Visited the computer laboratory (in the past two weeks combined)				
Yes	83 (51.2)	79 (48.8)	3.492	0.062
No	201 (42.8)	269 (57.2)		
Restriction of Internet use in school settings only				
Yes	66 (44.9)	81 (55.1)	0.000	0.991
No	218 (44.9)	267 (55.1)		
Restriction of Internet access both at home and in school settings				
Yes	34 (45.3)	41 (54.7)	0.005	0.941
No	250 (44.9)	307 (55.1)		
Self-perception of the presence of internet addiction				
Yes	130 (62.5)	78 (37.5)	38.651	<0.001
No	154 (36.3)	270 (63.7)		

Determinants of Internet addiction among adolescents

Adolescents in the mixed school had two times the odds of developing IA compared with those in the boys-only school (adjusted odds ratio (AOR) = 2.108, 95% confidence interval (CI) = 1.339-3.319, p ≤ 0.001). In-school adolescents in the girls-only school had nearly three times the odds of developing IA compared with those in the boys-only school (AOR = 2.675, 95%CI = 1.580-4.529, p = 0.001). In-school adolescents who did not access the Internet from both home and school settings had nearly 31% fewer odds of developing IA compared with their counterparts who accessed the Internet via multiple settings (AOR = 0.691, 95%CI = 0.481-0.991, p = 0.044) (Table 5).

**Table 5:** Determinants of internet addiction among in-school adolescents in Ibadan Southeast LGA, 2021

Variable	Categories	AOR	95% confidence interval		p-value
			Lower	Upper	
Age (years)	≤15	1	-	-	-
	>15	1.278	0.862	1.896	0.222
School	Boys’ only	1	-	-	-
	Mixed	2.240	1.434	3.498	<0.001
	Girls’ only	2.716	1.624	4.544	0.001
Current ownership of an Internet-enabled device	Yes	1.189	0.817	1.729	0.366
	No	1	-	-	-
Accessed the Internet from both home and school in the past one week	Yes	1	-	-	-
	No	0.710	0.497	1.014	0.060
Accessed the Internet in school only in the past one week	Yes	1.146	0.773	1.698	0.499
	No	1	-	-	-
Accessed the Internet at home only in the past one week	Yes	0.712	0.512	0.990	0.043
	No	1	-	-	-
Overall average time spent on the Internet weekly	≤3	1	-	-	-
	4–6	1.993	1.348	2.947	0.001
	>6	3.424	1.937	6.053	<0.001
Self-perception of problematic Internet use	Yes	2.338	1.610	3.397	<0.001
	No	1	-	-	

## Discussion

From this study, we identified that nearly 50% of in-school adolescents had IA. The high proportion obtained in this study could be explained as an aftermath of the COVID-19 lockdown restrictions that mandated physical isolation and compulsory homestay for at least five months in Nigeria and other parts of the globe [18,19]. The COVID-19 lockdown lasted from March 2020 to September 2020, and during this period, the only avenue through which many individuals could keep connected to the world was the Internet. Although this study was conducted a few months after all schools had reopened and were bound to observe COVID-19 safety measures including social distancing, many students and older individuals were yet to recover from the impacts of the COVID-19 lockdown. Therefore, such uncontrolled Internet connection during the lockdown could have transcended into IA even among in-school adolescents, long after the restrictions were lifted. The proportion of problematic Internet users found in this study is higher than the results of research conducted in Iran (40.7%), Turkey (9.7%), and Dhaka (24%) [20,21]. Our finding in the present study is similar to the 56.5% prevalence of IA reported from a study conducted among undergraduate students from a Malaysian public university [22].

This study found that Internet access from both school and home settings was a significant predictor of IA among adolescents. Given that adolescents spend more than half of each day in their homes, self-control needs to be practiced by these adolescents. In addition, parental control of adolescents’ phones and Internet access is noteworthy for preventing IA among adolescents. However, parents need to be cautious while weighing the different methods to enhance compliance with Internet use among their adolescents. The restrictive parenting approach has been established to have a positive association with IA among adolescents [23,24]. In many instances, the more the Internet control rules, the more likely it is that adolescents will become problematic Internet users. Therefore, the use of the restrictive parenting approach may be counterproductive in the context of IA among adolescents and may prompt adolescents to act in deviant ways while coping with stressful and unpleasant situations.

This study revealed that nearly 78% of in-school adolescents in Ibadan use Facebook more frequently than other social network types. The possible reasons for this observation are not far-fetched. Firstly, Facebook provides user-friendly services at lower costs compared to other social network types [25]. From their research, Ngonso et al. reported that a high proportion of Nigerian youths frequently accessed Facebook than other social network sites [26]. Facebook is the oldest social network site, and this could therefore provide some explanation on its high proportion of coverage. A cross-sectional study conducted among teenagers who are currently enrolled in high school has reported that although Facebook remains the most popular site accessed, teenagers commit to more than one social network platform [27]. Teenagers maintain a “social media portfolio” where top sites such as Facebook, Twitter, and Instagram are visited [27,28]. To prevent a situation where in-school adolescents invest too much time on social network sites to the detriment of their physical and mental health, the controlled use of these sites should be encouraged among adolescents.

The three Ms of parental involvement elucidated by the New York Center for Living would be important to yield optimal results in ensuring healthy Internet use among adolescents [29]. These would include parental modeling, through demonstration of the appropriate use of Internet-enabled devices; parental monitoring, such as the use of lights out and technology-off hours; and parental mentoring. Parental monitoring would entail that parents are engaged in their children’s world by building their interest in non-digital affairs and creating a robust offline schedule for exercise, parent/child activities, and family meditation.

This study revealed that Internet access in school could indeed be a potential gateway to the development of IA among in-school adolescents. Because adolescents spend nearly eight hours in school, teachers are required to monitor adolescents’ access to the computer laboratory, as well as activities engaged in during computer classes. Restricting Internet access from home and school settings should be undertaken to reduce the likelihood of the development of IA among adolescents. Likewise, adolescents should be discouraged from bringing phones to school, as this distracts them from the primary responsibility of learning.

Limitations

Many schools lack computer laboratories in Ibadan; thus, the sampling frame was restricted to a list of only a few schools. Because it was conducted in only one city, this study could lack some generalizability. The heterogeneous population of students in the schools enlisted in this study provides some generalizability to the general population of in-school adolescents. Despite these potential limitations, this study provides concrete evidence on the pattern of IA among in-school adolescents.

## Conclusions

The prevention of IA among in-school adolescents is multifaceted and requires a multidisciplinary approach. For effectiveness and sustainability on a long-term basis, many factors come to the fore: individual (adolescent), parental, school, and media. Therefore, we recommend the adoption of the following measures, especially during lockdown periods. Firstly, self-control skills such as self-monitoring and self-regulation of screen time (time spent on all Internet-enabled devices) should be adopted by adolescents themselves. Secondly, for the effectiveness of monitoring of Internet use, parents are required to act as role models; i.e., when they regulate their own pattern (frequency of access and time spent online) of Internet use, controlled Internet use can be enhanced among adolescents. Parents are also required to actively engage with adolescents during Internet activities such as knowing the different social media platforms that exist; decisions on adaptive Internet use could be jointly made by adolescents and their parent(s). Thirdly, school-based health awareness programs should be conducted on a regular basis on the dangers of IA and the need to regulate adolescents’ Internet use.

## Appendices

Table 6 shows the questionnaire used for data collection.

**Table 6:** Questionnaire

Part A: Sociodemographic Characteristics	
1. Age (in years as at last birthday): …………………………………………	
2. Sex:	
	Male
	Female
3. Who do you live with?	
	1. Parent
	2. Guardian
	3. Grandparents
	4. Siblings
	5. Others (please specify): …………………………………………
If you did not tick option 1 in question 3 above, skip to question 10.	
If you ticked option 1 in question 3 above, go to question 4.	
4. How old is your father?	
	1. Less than 40 years
	2. Between 40 and 60 years
	3. 60 years and above
5. How old is your mother?	
	1. Less than 40 years
	2. Between 40 and 60 years
	3. 60 years and above
6. What is your father’s highest educational qualification?	
	1. No formal education
	2. Primary
	3. Secondary
	4. Tertiary
	5. Postgraduate
7. What is your mother’s highest educational qualification?	
	1. No formal education
	2. Primary
	3. Secondary
	4. Tertiary
	5. Postgraduate
8. What is your father’s occupation? (please specify): …………………………………………	
9. What is your mother’s occupation? (please specify): …………………………………………	
10. What is the age range of the person you live with?	
	1. Less than 40 years
	2. Between 40 and 60 years
	3. 60 years and above
11. What family type do you currently live with?	
	1. Nuclear
	2. Extended
12. Among the people you live with, how many are 18 years and above?	
Part B: Pattern of Internet Use	
13. At what age (in years) did you first use the Internet?	
14. Do you currently own an Internet-enabled device?	
	1. Yes
	2. No
If No to question 14, skip to question 16.	
If Yes to question 14, go to question 15.	
15. Which of these devices do you currently own? (Tick all that apply.)	
	1. Smartphone
	2. Desktop
	3. Laptop
	4. Tablet
	5. Gaming device
	6. Others (please specify): …………………………………………
16. Which of the following devices have you used in the past one week to access the Internet? (Tick all that apply.)	
	1. Smartphone
	2. Desktop
	3. Laptop
	4. Tablet
	5. Others (please specify): …………………………………………
17. In the past one week, where have you connected to the Internet? (Tick all that apply.)	
	1. Home
	2. School
	3. Free public hotspots
	4. Paid hotspots
	5. Personal phone
	6. Others (please specify): …………………………………………
18. If No to question 14, who provided the device that you used to connect to the Internet? (Tick all that apply.)	
	1. A friend
	2. My parent(s)
	3. Others (please specify): …………………………………………
19. How often do you have access to the devices in question 18	
	1. Once a week
	2. 2–3 times weekly
	3. More than three times weekly
20a. Does your school have a computer laboratory?	
	1. Yes
	2. No
If No to question 20a, skip to question 25.	
20b. If Yes to question 20a, are there rules to student’s use of the computer in your school?	
	1. Yes
	2. No
21.	If Yes to question 20b, list the rules guiding student’s use of the computer in your school: ………………………………………………………………………………………………………………………………………………………………………………………………
If No to question 20b, skip to question 25.	
22. In the past two weeks combined, did you visit the computer laboratory?	
	1. Yes
	2. No
If No to question 22, skip to question 25.	
23. If Yes to question 22, how many hours did you spend in the computer laboratory?	
	1. Less than one hour
	2. 2–3 hours
	3. 3–4 hours
	4. More than four hours
24. During weekdays (Mondays to Fridays combined), how many hours did you spend on the Internet in the school in the past two weeks?	
	1. Nil
	2. Less than one hour
	3. 2–3 hours
	4. 3­–4 hours
	5. More than four hours
25. After returning home from school (Mondays to Fridays combined), how many hours did you averagely spend on the Internet in the past one week?	
	1. Less than one hour
	2. 2–3 hours
	3. 3–4 hours
	4. More than four hours
26. During weekends (Saturdays and Sundays combined), how many hours did you spend on the Internet in the past one week?	
	1. Less than one hour
	2. 2–3 hours
	3. 3–4 hours
	4. More than four hours
27. What are your reasons for going online? (Tick all that apply.)	
	1. Communication
	2. Socialization
	3. School assignment
	4. Information research
	5. Others (please specify): …………………………………………
28. What have you done online in the past 24 hours?	
	1. Watching movies/serials/videos
	2. Downloads
	3. Reading magazines/books
	4. Listening/watching music
	5. Online gaming
	6. Chatting with friends
	7. Using e-mails
	8. Making phone calls
	9. Internet social network
29. Which Internet social network type have you used in the past one week? (Tick all that apply.)	
	1. Facebook
	2. WhatsApp
	3. Instagram
	4. Snapchat
	5. Twitter
	6. YouTube
	7. Others (please specify): …………………………………………
30. Are your parent/guardian (in question three above) aware that you use the Internet?	
	1. Yes
	2. No
31. Has anyone ever told you that excessive Internet use could be problematic for you?	
	1. Yes
	2. No
If No to question. 31, skip to question 34.	
32. If Yes to question 31, who are those people? (Tick all that apply.)	
	1. Parent
	2. Guardian
	3. Teacher
	4. Others (please specify): …………………………………………
33. How have they enforced that you do not excessively use the Internet? (Tick all that apply.)	
	1. Checking my browsing device
	2. Frequent reminder to be careful
	3. Strict monitoring of the Internet
	4. Fixing specific browsing time
	5. Others (please specify): …………………………………………
34. Do you think you are addicted to the Internet?	
	1. Yes
	2. No
If No to question 34, skip to question 36.	
35. If Yes to question 34, why do you think you have problematic Internet use? ………………………………………………………………………………………………………………………………………………………………………………………………………………………………………………………………………………………………………………	
36. What are the causes of problematic Internet use? (Tick all that apply.)	
	1. Loneliness
	2. Boredom
	3. Idleness
	4. Physical isolation
	5. Parental neglect
	6. Conflict with family members
	7. Parental Internet addiction
	8. Low self-esteem
	9. Poor physical relationship
	10. Stress
37. What are the effects of Internet addiction? (Tick all that apply.)	
	1. Loss of sleep
	2. Anxiety
	3. Depression
	4. Reduced physical relationships
	5. Family disconnectedness
	6. Thoughts of an empty life
	7. Reduced work productivity
	8. Procrastination
	9. Secrecy/defensiveness
	10. Neglect of household chores
38. How can you prevent problematic Internet use? (Tick all that apply.)	
	1. Personal discipline
	2. Parental control of Internet use
	3. Parental control of phone use
	4. Reducing time spent in the computer laboratory
	5. Closing the computer laboratory when it is not used
	6. Improving family relationship

S/N	Questions	Never	Once weekly	Twice weekly	Three times weekly	Four times weekly	Five times weekly or more
1.	How often do you stay online longer than you intend?						
2.	How often do you neglect household chores to spend more time online?						
3.	How often do you lose sleep due to late night log-ins?						
4.	How often do you try to hide how long you have been online?						
5.	How often do you feel depressed, moody, or nervous when you are offline, which go away when you are back online?						
6.	How often do your school grades suffer because of the amount of time you spend online?						
7.	How often do your job performance or productivity suffer because of the Internet?						
8.	How often do you become defensive or secretive when anyone asks you what you do online?						
9.	How often do you check your e-mail before something else that you need to do?						
10.	How often do you find yourself anticipating when you will go online again?						
11.	How often do others in your life complain to you about the amount of time you spend online?						
12.	How often do you find yourself saying “just a few minutes more” when online?						
13.	How often do you try to cut down the amount of time you spend online and fail?						
14.	How often do you prefer the excitement of the Internet to intimacy with your family?						
15.	How often do you form new relationships with fellow online users?						
16.	How often do you block out disturbing thoughts about your life with soothing thoughts of the Internet?						
17.	How often do you fear that life without the Internet would be boring, empty, and joyless?						
18.	How often do you snap, yell, or act annoyed if someone bothers you while you are online?						
19.	How often do you feel preoccupied with the Internet when offline or fantasize about being online?						
20.	How often do you choose to spend more time online over going out with others?						
